# 异基因造血干细胞移植后序贯阿伐替尼治疗系统性肥大细胞增多症伴RUNX1-RUNX1T1阳性急性髓系白血病2例并文献复习

**DOI:** 10.3760/cma.j.cn121090-20240313-00092

**Published:** 2024-05

**Authors:** 娟 王, 璎玲 祖, 瑞瑞 桂, 珍 李, 䶮莉 张, 健 周

**Affiliations:** 郑州大学附属肿瘤医院，河南省肿瘤医院血液科，郑州 450008 Department of Hematology, Henan Cancer Hospital, The Affiliated Cancer Hospital of Zhengzhou University, Zhengzhou 450008, China

## Abstract

系统性肥大细胞增多症（SM）伴RUNX1-RUNX1T1阳性急性髓系白血病（AML）是一种较为罕见的髓系肿瘤，目前尚无标准的治疗方案，河南省肿瘤医院异基因造血干细胞移植（allo-HSCT）序贯阿伐替尼治疗2例SM伴RUNX1-RUNX1T1阳性AML患者，骨髓中肥大细胞消失，C-KIT突变和RUNX1-RUNX1T1融合基因持续阴性。提示allo-HSCT后序贯阿伐替尼可作为SM伴RUNX1-RUNX1T1阳性AML患者的有效治疗手段。

## Abstract (en)

Systemic mastocytosis（SM）with RUNX1-RUNX1T1 positive acute myeloid leukemia（AML）is a rare myeloid tumor with no standard treatment. Two cases of SM patients with RUNX1-RUNX1T1 positive AML treated with sequential avapritinib after allogeneic hematopoietic stem cell transplantation（allo-HSCT）were reported in Henan Cancer Hospital. Mast cell in bone marrow disappeared, C-KIT mutation and RUNX1-RUNX1T1 fusion gene remained negative. Allo-HSCT sequential avapritinib is an effective treatment for SM patients with RUNX1-RUNX1T1 positive AML.

系统性肥大细胞增多症（Systemic mastocytosis, SM）是侵袭性肥大细胞在真皮组织以外的一个或多个组织、器官或系统的克隆性增殖，并释放大量的血管活性介质，引起多器官功能障碍的一种异质性疾病[1]，SM伴血液系统肿瘤（SM with associated hematologic neoplasm, SM-AHN）是其中一种亚型。SM-AHN临床较为罕见，国内少有报道，其临床表现和预后存在很大异质性，中位总生存时间约为24个月[2]，其中SM伴RUNX1-RUNX1T1阳性急性髓系白血病（Acute myeloid leukemia, AML）患者的预后极差。现报道我院异基因造血干细胞移植（allo-HSCT）后序贯阿伐替尼治疗2例SM伴RUNX1-RUNX1T1阳性AML患者，并复习相关文献，以提高对此类疾病诊疗的认识。

## 病例资料

例1，男，18岁，无明显诱因出现发热，最高体温38.9 °C，伴乏力，当地医院查血常规：WBC 10.5×10^9^/L、HGB 49 g/L、PLT 8×10^9^/L，2021年7月25日入我院诊治。

入院查体：重度贫血貌，双下肢可见散在出血点，全身浅表淋巴结无肿大，胸骨无压痛，心肺听诊无异常，肝脾肋缘下未触及。骨髓象：增生明显活跃，原始粒细胞占0.100，肥大细胞占0.336；外周血涂片原始粒细胞占0.460，肥大细胞占0.020。骨髓免疫分型：异常髓系原始细胞占0.116，肥大细胞占0.040，该群肥大细胞高表达CD117、表达CD25、弱表达CD2。骨髓病理：增生极度活跃，原始幼稚细胞多见，肥大细胞广泛增生（约70.0％）。染色体核型：45, X, −Y, t（8;21）（q22;q22）[18]/46, XY[2]。白血病融合基因筛查：RUNX1-RUNX1T1阳性。白血病108个基因相关基因突变：KIT-D816V阳性，基因突变频率18.8％。诊断：SM伴RUNX1-RUNX1T1阳性AML。

7月26日起给予IA方案（伊达比星20 mg/d，第1～3天；阿糖胞苷200 mg/d，第1～7天）诱导化疗。诱导结束第28天骨髓象：增生明显活跃，原始粒细胞占0.020，肥大细胞占0.360，散在或成簇状分布。流式细胞术检测微小残留病（Minimal residual desease, MRD）：异常髓系原始细胞占0.007，肥大细胞占0.156。患者白血病获得完全缓解（Complete remission, CR），接受2次大剂量阿糖胞苷联合达沙替尼巩固化疗。化疗期间共行4次腰椎穿刺+三联鞘注预防中枢神经系统白血病。该患者MRD持续阳性，肥大细胞持续存在，排除移植禁忌证，行allo-HSCT，移植前骨髓象：有核细胞增生明显活跃，原始粒细胞占0.016，肥大细胞占0.400，散在或聚集分布。MRD：异常髓系原始细胞占0.005，肥大细胞占0.168。预处理方案为改良Bu/Cy：司莫司汀（450 mg，−6 d）、阿糖胞苷（3 g每12 h 1次，−6～−5 d），白消安（45 mg每6 h 1次，−6～−4 d），环磷酰胺（3 g/d，−3～−2 d），抗人T细胞兔免疫球蛋白（5 mg·kg^−1^·d^−1^，−4～−1 d）。2021年11月30日回输无关供者HLA 10/10相合外周血干细胞（单个核细胞18.503×10^8^/kg，CD34^+^细胞9.081×10^6^/kg）。予环孢素A、吗替麦考酚、甲氨蝶呤预防移植物抗宿主病（Graft versus-host disease, GVHD）。+18 d粒细胞植入，+20 d血小板植入。+28 d复查骨髓象：有核细胞增生明显活跃；未见原幼粒细胞，肥大细胞占0.156；MRD：髓系原始细胞占0.004，肥大细胞占0.141。嵌合率：73.88％。C-KIT基因突变频率15.3％；RUNX1-RUNX1T1/ABL 11.4％。免疫抑制剂减量。+42 d复查骨髓象：有核细胞增生明显活跃；未见原幼粒细胞，肥大细胞占0.118；MRD：未见异常原始细胞，肥大细胞占有核细胞0.021。嵌合率：68.81％。给予阿伐替尼100 mg/d靶向治疗。+56 d复查骨髓象：有核细胞增生减低，未见原幼粒细胞，肥大细胞占0.050；MRD：未见异常原始细胞，肥大细胞占0.015。嵌合率：87.86％。期间合并2级骨髓抑制，阿伐替尼减量为50 mg/d，血象逐渐恢复正常。+84 d复查骨髓象和MRD均未见异常原始细胞和肥大细胞。C-KIT突变和RUNX1-RUNX1T1/ABL均阴性。嵌合率：100.00％。阿伐替尼治疗1年后停药。随访至2024年2月29日，期间规律复查，患者持续处于CR状态，MRD和基因检测均阴性，嵌合体为完全嵌合状态，已停用免疫抑制剂，目前无病生存中。

例2，女，57岁，受凉后发热，最高体温38.2°C，伴乏力、咳嗽、咳痰，当地医院查血常规：WBC 5.24×10^9^/L、HGB 76 g/L、PLT 5×10^9^/L，骨髓象：有核细胞增生极度活跃，原始粒细胞占0.556。骨髓免疫分型：原始/幼稚髓系细胞占0.680。骨髓病理：有核细胞增生过度；偏幼稚细胞弥漫增生。染色体核型：46, XX, t（8;21）（q22;q22）[20]。白血病融合基因筛查：RUNX1-RUNX1T1阳性。诊断为AML，2022年8月8日转至我院。

入院查体：贫血貌，全身散在瘀斑，全身浅表淋巴结无肿大，胸骨无压痛，心肺听诊无异常，肝脾肋缘下未触及。2022年8月10日给予IA方案诱导化疗，诱导第4天白血病相关基因突变检测结果回示KIT-D816V阳性，基因突变频率38.02％。加用达沙替尼50 mg/d靶向治疗，应用达沙替尼第2天出现胸闷，端坐呼吸，双肺可闻及湿啰音，胸部CT表现为两肺纹理增粗模糊，呈毛玻璃样改变，可见Kerley B线，双侧胸腔少量积液。考虑达沙替尼所致肺水肿，停药并给予呋塞米利尿等治疗后症状缓解。诱导结束后第22天复查骨髓象：增生明显活跃，原始粒细胞0.008。肥大细胞0.264，散在或聚集分布。MRD：未见异常原始髓系细胞，肥大细胞占0.108，表达CD117、CD30、CD25及CD2。骨髓活检：骨髓增生活跃，可见肥大细胞局灶性致密浸润，占骨髓细胞10.0％以下。修正诊断：SM伴RUNX1-RUNX1T1阳性AML。

患者接受2次大剂量阿糖胞苷、3次维奈克拉联合阿扎胞苷方案化疗，化疗期间共行4次腰椎穿刺+三联鞘注预防中枢神经系统白血病。骨髓象均为CR状态，但肥大细胞持续存在且RUNX1-RUNX1T1定量处于较高水平，排除移植禁忌证，行allo-HSCT，移植前骨髓象：增生活跃，未见原粒细胞，肥大细胞占0.036。MRD：未见异常原始细胞，肥大细胞占有核细胞0.072。染色体为正常核型，RUNX1-RUNX1T1/ABL 39.63％；KIT-D816V基因突变频率5.36％。预处理方案：氟达拉滨50 mg/d、−6～−2 d；白消安180 mg/d、−6～−4 d；司莫司汀500 mg、−3 d；马法兰154 mg、−2 d；抗人T细胞兔免疫球蛋白5 mg·kg^−1^·d^−1^、−4～−1 d。2023年3月29日回输无关供者HLA 10/10相合外周血干细胞（单个核细胞9.982×10^8^/kg，CD34^+^细胞4.191×10^6^/kg），予以移植后环磷酰胺（PT/CY）、环孢素A、芦可替尼及霉酚酸酯预防GVHD，+12 d粒细胞及血小板植入。+28 d复查骨髓象：有核细胞增生活跃，未见原幼粒细胞和肥大细胞。MRD：未见异常原始细胞，肥大细胞占0.183。嵌合率94.28％，C-KIT基因突变频率2.94％；RUNX1-RUNX1T1/ABL 2.30％；给予阿伐替尼100 mg/d靶向治疗，免疫抑制剂减量。+56 d复查骨髓象和MRD均未见异常原始细胞和肥大细胞。嵌合率99.67％，C-KIT突变和RUNX1-RUNX1T1/ABL均阴性。合并2级骨髓抑制，阿伐替尼减量为50 mg/d。+70 d合并3级骨髓抑制，停用阿伐替尼，+84 d骨髓抑制降至1级，后因个人原因未继续服用阿伐替尼。随访至2024年2月29日，期间规律复查，患者持续处于CR状态，MRD和基因检测均阴性，嵌合体为完全嵌合状态，已停用免疫抑制剂，目前无病生存中。

## 讨论及文献复习

SM-AHN是指SM伴AML、骨髓增生异常综合征、非霍奇金淋巴瘤等克隆性血液病[3]，约占SM的12.1％[4]。SM-AHN的诊断需要同时符合SM和血液肿瘤的诊断标准。但部分患者初诊时肥大细胞浸润可能被肿瘤细胞浸润掩盖，因此对于伴有KIT-D816V突变的髓系肿瘤患者可在细胞消减后再次进行SM的诊断。

临床表现方面，SM可以有全身症状（发热、疲劳、体重减轻、盗汗等）、肥大细胞活化症状（介质导致的症状）、相关疾病症状（血液系统疾病相关症状等）以及体征（肝脾肿大和淋巴结肿大等）。

SM的治疗主要分为3个方面[2]–[3],[5]，分别为针对肥大细胞活化症状的抗介质治疗（白三烯抑制剂、H1/2受体拮抗剂等）、针对器官损伤的治疗（抗骨质疏松、改善胃肠功能等）和针对肥大细胞的治疗（传统化疗药物、靶向治疗、allo-HSCT等）。一项国际多中心回顾性临床研究显示，SM-AHN患者接受allo-HSCT后3年总生存率为74％[6]。Pullarkat等[7]回顾性分析10例SM合并AML-ETO融合基因阳性AML患者，仅接受allo-HSCT的2例患者长期生存。张玉洁等[8]报道应用allo-HSCT治疗2例AML-M2伴SM患者，至随访终点，均为CR状态。

本文2例患者移植后早期虽处于CR状态，但异常肥大细胞仍存在、C-KIT突变及RUNX1-RUNX1T1基因阳性提示复发风险极高。移植物抗白血病效应可预防白血病复发，但无关供者allo-HSCT早期快速减停免疫抑制剂可诱发严重的急性GVHD。考虑SM-AHN患者伴有KIT突变，在治疗中可联合靶向KIT的小分子激酶抑制剂。临床中常用的靶向药物有伊马替尼、达沙替尼、米哚妥林、阿伐替尼等。伊马替尼于2006年被FDA批准用于治疗伴野生型KIT或未知突变状态的成人侵袭性系统性肥大细胞增多症患者。Alvarez-Twose等[9]–[10]研究显示KIT-D816V阴性高分化良好的SM患者，应用伊马替尼治疗12个月，总体反应率为50％。但伊马替尼对KIT-D816V突变缺乏活性，因此不适用于伴KIT-D816V突变的SM患者。达沙替尼对野生型及KIT-D816V位点突变均有抗肿瘤活性[11]，但部分患者治疗效果差或难以耐受达沙替尼不良反应。米哚妥林对KIT-D816V和KIT-D816Y突变以及FLT3突变具有体外活性，2016年在美国被批准用于SM-AHN的治疗。Gotlib等[12]的一项开放性研究入组了57例SM-AHN患者，米哚妥林治疗有效率为58％，疗效中位持续时间为12.7个月，中位总生存时间为20.7个月，至随访终点，中位无进展生存时间为11.3个月，具有良好的安全性。阿伐替尼是一种强效的Ⅰ型多激酶抑制剂，对KIT活化环区域突变具有选择性活性[13]，被FDA批准用于治疗成人进展型SM患者。PATHFINDER研究[14]对32例接受阿伐替尼治疗的SM患者进行了中期分析，中位随访10.4个月，确认的总有效率为75％。目前尚无针对阿伐替尼和其他可用治疗方法正面比较的报道。一项全球多中心回顾性研究[15]收集了接受最佳可用治疗（best available therapy, BAT）的进展型系统性肥大细胞增多症（advanced systemic mastocytosis, AdvSM）患者的数据，包括176例阿伐替尼患者和141例BAT患者，BAT组50％的患者曾接受米哚妥林治疗，25％的患者曾接受克拉屈滨治疗。研究结果显示，阿伐替尼组治疗持续时间更长（30.6个月对5.5个月），中位总生存时间明显延长（49.0个月对26.8个月）。体外研究发现，阿伐替尼对KIT-D816V的抑制效果明显优于米哚妥林[16]–[17]。Reiter等[18]的研究显示阿伐替尼治疗SM-AHN有效率为77％（17/22），明显高于米哚妥林。且相关研究显示阿伐替尼具有良好的安全性[19]–[20]。

本研究2例患者在移植后均接受阿伐替尼靶向治疗，同时逐渐减量免疫抑制剂，例1在治疗42 d后、例2在治疗28 d后复查骨髓象和MRD均未见肥大细胞，C-KIT突变和RUNX1-RUNX1T1/ABL均阴性，嵌合体均为完全嵌合。截至随访终点，2例患者无病生存时间分别为26.3、10.0个月。应用阿伐替尼期间2例患者分别出现2级和3级血液学不良反应，减量或停药后可恢复或降级，未观察到非血液学不良反应。

综上所述，allo-HSCT后序贯阿伐替尼可作为SM伴RUNX1-RUNX1T1阳性AML有效的治疗手段，主要不良反应为血液学不良反应。
